# Noninvasive brain stimulation for migraine: A bibliometric analysis of scientific research with thematic and keyword mapping

**DOI:** 10.1097/MD.0000000000051349

**Published:** 2026-10-30

**Authors:** Turki Abualait, Shahid Bashir, Mohamed A. Al Maghraby, Muhammad Ajmal Khan, Nadeem Siddique, Suzan Ahmed Al-Hamadah, Saliha Mohammed Alaali, Zahra Abdulmuttalib Al-Muhanna, Danah Khalid Al-jaafar, Lamia Ali Al-salman, Rama Emad Shabana, Zahra Hadi Alqallaf

**Affiliations:** a College of Applied Medical Sciences, Imam Abdulrahman Bin Faisal University, Dammam, Saudi Arabia; b Neuroscience Center, King Fahad Specialist Hospital, Dammam, Saudi Arabia; c King Salman Center for Disability Research, Riyadh, Saudi Arabia; d Directorate of Library Affairs, Imam Abdulrahman Bin Faisal University, Dammam, Saudi Arabia; e Library Department, Lahore University of Management Sciences, Lahore, Pakistan.

**Keywords:** author keywords, bibliometric analysis, keyword co-occurrence, migraine, noninvasive brain stimulation, thematic mapping

## Abstract

**Background:**

Migraine is a leading cause of global disability, with noninvasive brain stimulation (NIBS) emerging as a promising therapeutic alternative. Despite growing research, no bibliometric study has synthesized the field’s evolution, collaboration patterns, thematic trajectories, knowledge gaps and keyword-based research frontiers.

**Methods:**

We analyzed 411 publications (1992–2024) from the Web of Science Core Collection using performance analysis, science mapping (VOSviewer, CiteSpace), and thematic and keyword co-occurrence analysis using science mapping software tool. Metrics included productivity, citations, collaboration networks, author keyword frequency, co-occurrence clustering, and citation-burst detection.

**Results:**

Annual publications grew at 11.75%, peaking post-2013 (Food and Drug Administration approval of transcranial magnetic stimulation for migraine). The United States led in output (33.1%), while Denmark had the highest citation impact (93.1 citations/publication). Keyword analysis revealed 6 thematic clusters: pathophysiology (e.g., cortical excitability), clinical subtypes (e.g., chronic migraine), and emerging therapies (e.g., vagus nerve stimulation [VNS]). Citation-burst detection identified cortical excitability (2000–2014) and noninvasive VNS (2018–2024) as key turning points. European institutions were prominent within the collaboration networks, indicating substantial inter-institutional and international research connectivity.

**Conclusions:**

This bibliometric analysis maps the evolution, collaboration patterns, thematic structure, and emerging research priorities of noninvasive brain stimulation research in migraine, highlights transcranial magnetic stimulation and transcranial direct current stimulation as established methods, with noninvasive VNS as an emerging frontier, while identifying geographic and thematic gaps that may inform future research.

## 1. Introduction

Migraine is a widespread and disabling neurological disorder, impacting approximately 15% of the global population and ranking as the second-leading cause of disability worldwide.^[1]^ While pharmacological treatments, including triptans and calcitonin gene-related peptide inhibitors, have seen notable progress, a significant proportion of patients continue to experience inadequate relief or intolerable side effects.^[2]^ This persistent therapeutic gap has spurred interest in non-pharmacological therapeutic approaches, which offer alternatives that avoid systemic drug exposure and can be tailored to individual patient profiles. Among these approaches, noninvasive brain stimulation (NIBS) techniques, including transcranial magnetic stimulation (TMS), transcranial direct current stimulation (tDCS), vagus nerve stimulation (VNS), and emerging modalities such as photobiomodulation, have gained increasing attention for their potential to modulate cortical excitability, disrupt pain pathways, and reduce migraine frequency and severity, without systemic drug effects.^[3,4]^

There is robust evidence in the literature that TMS and tDCS have evolved from experimental tools to clinically feasible options within the NIBS field.^[5,6]^ Evidence highlights the transition of TMS to routine clinical use for several neurological and neuropsychiatric indications.^[7]^ tDCS, while not yet as widely approved for clinical use, is recognized as an emerging intervention with growing evidence for efficacy in neurological conditions and is increasingly being evaluated in clinical trials.^[8,9]^ A pivotal moment came in 2013 when the United States (US) Food and Drug Administration (FDA) approved single-pulse TMS (sTMS) for migraine with aura, signaling growing recognition of neuromodulation as a legitimate therapeutic strategy.^[10]^ Despite an expanding body of clinical trials and narrative reviews, the broader research background, encompassing collaboration patterns, geographic contributions, and thematic evolution, remains unknown.

Bibliometric analysis offers a powerful means to address this gap.^[11,12]^ By examining publication trends, authorship networks, and keyword dynamics, such studies illuminate not only research priorities but also the pathways through which knowledge is disseminated. Prior bibliometric work in neurology has highlighted disparities in global research output^[11]^ and delays in translating preclinical findings into clinical practice.^[12]^ Yet, no study has applied this approach specifically to NIBS research in migraine, leaving critical questions unanswered: Which institutions and countries are driving innovation? How have research themes shifted over time? Where do the most pressing knowledge gaps lie?

Although previous bibliometric studies have examined individual neuromodulation modalities^[13–15]^ or broader neurological applications, a comprehensive bibliometric assessment integrating multiple NIBS modalities specifically within migraine research remains limited. The present study addresses this gap by combining publication performance, author and institutional productivity, international collaboration, keyword co-occurrence, citation-burst detection, and thematic evolution to characterize the development and emerging priorities of NIBS research in migraine.

## 2. Methods

### 2.1. Data source and search strategy

The dataset was retrieved from the Web of Science Core Collection (WoSCC) on May 10, 2025, covering peer-reviewed publications from 1992 to December 31, 2024. A comprehensive search query was constructed using Boolean operators to capture all relevant terminology related to migraine and NIBS, including TMS, tDCS, VNS, and other emerging modalities.

Boolean operators were used to connect the final keywords to formulate the research query. In 2025, the following query was executed in the search interface of the WoSCC:

TS = (Migraine*) AND TS=(“Transcranial Direct Current Stimulation” OR “tDCS” OR “Transcranial Alternating Current Stimulation” OR “tACS” OR “Transcranial Random Noise Stimulation” OR “tRNS” OR “Amplitude-Modulated Transcranial Electrical Stimulation” OR “AM-tES” OR “High-Definition tDCS” OR “HD-tDCS” OR “Cranial Electrotherapy Stimulation” OR “CES” OR “Transcranial Magnetic Stimulation” OR “TMS” OR “Repetitive Transcranial Magnetic Stimulation” OR “rTMS” OR “Intermittent Theta Burst Stimulation” OR “iTBS” OR “Continuous Theta Burst Stimulation” OR “cTBS” OR “Deep Transcranial Magnetic Stimulation” OR “dTMS” OR “Transcranial Rotating Permanent Magnet Stimulation” OR “TRPMS” OR “Transcranial Focused Ultrasound Stimulation” OR “tFUS” OR “Focused Ultrasound Stimulation” OR “FUS” OR “Low-Intensity Focused Ultrasound Stimulation” OR “LIFU” OR “Low-Intensity Focused Ultrasound Pulsation” OR “High-intensity focused ultrasound” OR “HIFU” OR “Transcranial Photobiomodulation” OR “tPBM” OR “Transcranial infrared laser stimulation” OR “TILS” OR “low-level laser therapy” OR “transcranial pulsed-current stimulation” OR “Transcranial Pulse Stimulation” OR “TPS” OR “Vagus nerve stimulation” OR “VNS” OR “Auricular vagus nerve stimulation” OR “aVNS” OR “transcutaneous auricular Vagus Nerve Stimulation” OR “taVNS” OR “External vagus nerve stimulation” OR “noninvasive vagus nerve stimulation” OR “nVNS” OR “Vagal neuromodulation” OR “Vagus nerve neuromodulation” OR “Cervical vagus stimulation” OR “Temporal Interference Stimulation” OR “Interferential stimulation” OR “temporal interference stimulation” OR “TI”).

### 2.2. Inclusion and exclusion criteria

The query resulted in 1175 records. The document types of Editorial Materials (27), Meeting Abstracts (162), Letters (18), Corrections (2), Reprint (1), Book Chapter (18), and Retracted Publications (6) were excluded from the research. Following that, 20 documents published in 2025 were also excluded. As a result, a total of 923 documents were downloaded in comma-separated values, research information systems, and bibliography formats. Two reviewers independently screened the titles, abstracts, and author keywords of these records using the predefined eligibility criteria. Records were considered eligible when they focused specifically on NIBS in the context of migraine and contained sufficient bibliographic information for bibliometric analysis. Records were excluded when NIBS was unrelated to migraine, migraine was not a relevant study population or outcome context, the publication concerned an intervention outside the predefined NIBS scope, or the record lacked sufficient relevance based on the title, abstract, and author keywords. Thus, 512 records were excluded as outside the predefined scope, leaving 411 records that included Article (296), Proceeding Paper (7), and Review (108) documents for the analysis, as shown in Figure 1.

**Figure 1.**
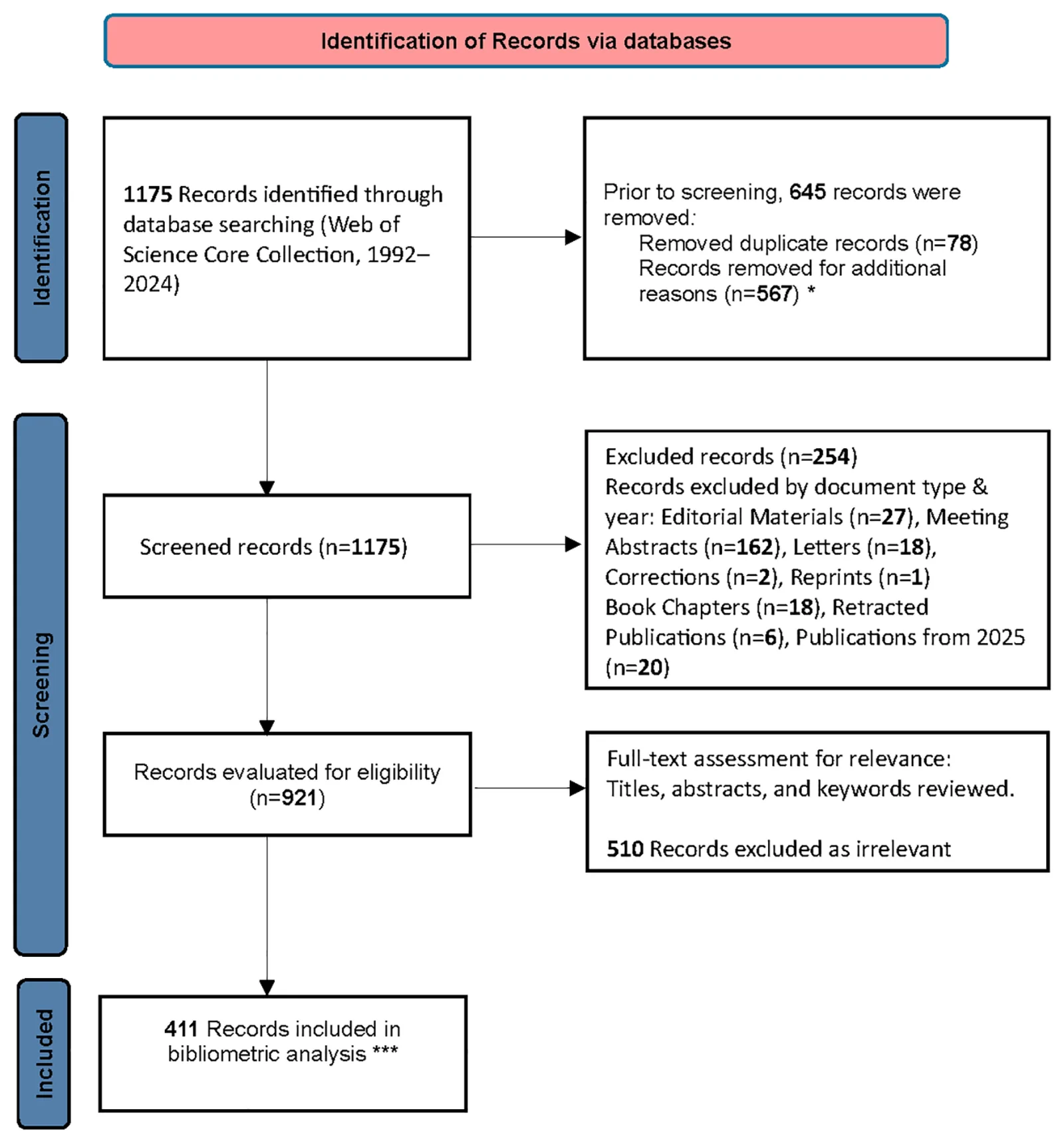
PRISMA flowchart for the bibliometric analysis. n = number of records, PRISMA = Preferred Reporting Items for Systematic reviews and Meta-Analyses.

The supplementary data file is provided in Excel format and contains a complete list of all 411 publications included in the analysis (File S2, Supplemental Digital Content 1). The raw WoSCC export files are not publicly deposited because access to the database and redistribution of exported records may be subject to Web of Science licensing restrictions. The bibliographic search strategy, inclusion/exclusion criteria, software versions, analytical parameters, and complete list of included publications are provided to facilitate reproducibility.

### 2.3. Data enrichment and keyword standardization

The data were imported into EndNote, a citation management tool, to identify and remove duplicates based on author, title, and year. No duplicates were detected, resulting in a total of 411 documents included in the current bibliometric analysis. To address the issue that some documents lacked author-supplied keywords, Biblioanalytics software was utilized,^[16]^ which combined all author-supplied keywords extracted from the titles, abstracts, and author keywords across all 411 documents. A keyword thesaurus was then constructed by identifying all terms that appeared more than 10 times and recording them individually. A minimum occurrence threshold of 10 was selected to retain recurrent and sufficiently represented keywords while reducing noise from infrequently occurring terms and producing a network of manageable density for visual interpretation. This step ensured semantic consistency and improved the reliability of co-occurrence and thematic analyses. Author country and organization were confirmed with the help of city (Affiliation) and reprint address (corresponding author address). The study utilized Microsoft Word, Excel, VOSviewer, CiteSpace, Biblioanalytics, Power BI, WordArt.com, Scimago Graphica, and Bibliomaster for data analysis and visualization.

## 3. Data analysis

The frequency was calculated using IBM SPSS Statistics (Version 24.0). The following information: yearly publication growth, document types, authorship pattern, most productive authors, organizations and countries, most prolific sources, top 10 keywords with the strongest citation bursts, co-occurrence of author keywords, word cloud of author keywords, co-authorship density visualization, and subject categories-wise evolution of research were extracted and analyzed.

Two authors independently reviewed the retrieved records based on title, abstract, and author keywords using the predefined eligibility criteria. Disagreements were initially resolved through discussion and consensus between the 2 reviewers. When consensus could not be reached, a third reviewer was consulted. The same eligibility criteria were applied consistently throughout the screening process. Inter-rater agreement was assessed using Cohen κ. Thus, only studies that specifically focused on NIBS techniques in migraine were included in the analysis. The final selection was based on the predefined eligibility criteria rather than citation counts. For each article, data were extracted on the title, authors, journal, citation count, publication year, country of origin, journal impact factor, article type, and Web of Science subject category. If the corresponding author had multiple affiliations from different countries, the first-listed affiliation was used to determine the country of origin. In cases where articles were classified under multiple subject categories, only the first category was considered. When multiple authors were listed, the highest-ranked author was designated as the first author, and the lowest-ranked as the last.

### 3.1. Keyword and thematic analysis

Keyword-based analysis was central to mapping the intellectual structure and evolution of the field. We employed a triangulated approach using 3 complementary bibliometric tools:

- VOSviewer v1.6.20 was used^[17]^ to construct a keyword co-occurrence network. The analysis included all author and indexed keywords, with a minimum occurrence threshold of 10. This threshold was selected to retain recurrent and sufficiently represented keywords while reducing noise from infrequently occurring terms and producing a network of manageable density for visual interpretation. Link strength was calculated using association strength, and clusters were identified using the VOS modularity-based clustering algorithm. This produced a visual map of 6 thematic clusters, each representing a distinct research domain. Formal sensitivity analyses using alternative keyword thresholds were not prespecified or conducted; therefore, the thematic structure should be interpreted in the context of the selected threshold of 10 occurrences.
- CiteSpace v6.1.R4 was applied^[18,19]^ to detect citation bursts in keywords using Kleinberg algorithm (*σ* = 12, *γ* = 1.0). This method identified keywords with sudden increases in citation frequency, revealing emerging or declining research fronts over time (e.g., “cortical excitability” during 2000–2014; “vagus nerve stimulation” during 2018–2024).
- Science mapping software tool v1.1.0 was used to trace the thematic evolution of the field across 3 time slices: 1992 to 2009, 2010 to 2020, and 2021 to 2024, periods defined by publication growth inflection points. Keyword similarity was measured using the Jaccard index, and thematic clusters were generated using the Simple Centers algorithm. This allowed us to track how research priorities shifted from pathophysiological mechanisms to clinical subtypes and, more recently, to novel neuromodulation therapies.

Additionally, a word cloud was generated using WordArt.com to visualize keyword frequency, with font size proportionate to occurrence count.

### 3.2. Performance and network analysis

Beyond keywords, we conducted performance analysis of countries, institutions, and authors, using total publications (TP) and citations per publication (C/P) as indicators of productivity and impact, respectively. Collaboration networks were mapped using VOSviewer (co-authorship) and CiteSpace (institutional and country co-occurrence), with a minimum link strength of 2 collaborative publications. Country affiliations were assigned based on the city (address) and reprint address (corresponding author address). For country-level productivity analyses, the first-listed affiliation/country was used as the primary country assignment to ensure a single, consistent country attribution for each publication and to avoid double counting. However, co-affiliated countries remained relevant to the collaboration-network analyses, and the use of a primary country assignment for productivity measures may underestimate the contribution of countries represented through secondary affiliations.

### 3.3. Validation and rreproducibility

Inter-rater reliability for relevance screening was high (Cohen κ = 0.89, 95% confidence interval: 0.82–0.94). Thematic cluster labels were validated through manual content review by 3 authors, based on core keywords and representative publications. All software versions, parameter settings, and analytical workflows are explicitly reported to ensure full reproducibility.

Figure S1, Supplemental Digital Content 2 provides a summary of the dataset, which is provided in the File S1, Supplemental Digital Content 9.

## 4. Results

### 4.1. Yearly publication growth

Figure 2 illustrates the yearly growth in publications related to the topic under study. The year 1992 witnessed the arrival of the first publication on the topic. The second publication appeared in 1996 after a gap of 3 years. The next 3 publications were contributed in 1998 after a 1-year gap. After 1998, research on the topic started to be published without any yearly gap. Up to 2005, only a single-digit number of documents were published on the topic. In 2006 and 2007, double-digit documents, that is, 12 and 16, were contributed. Again, in 2008 and 2009, only a single digit of documents was published. After 2009, the topic gained the attention of researchers, and a double-digit number of publications started appearing. The highest number of publications (n = 35) was published in 2024, followed by 2021 (n = 34) and 2022 (n = 22).

**Figure 2.**
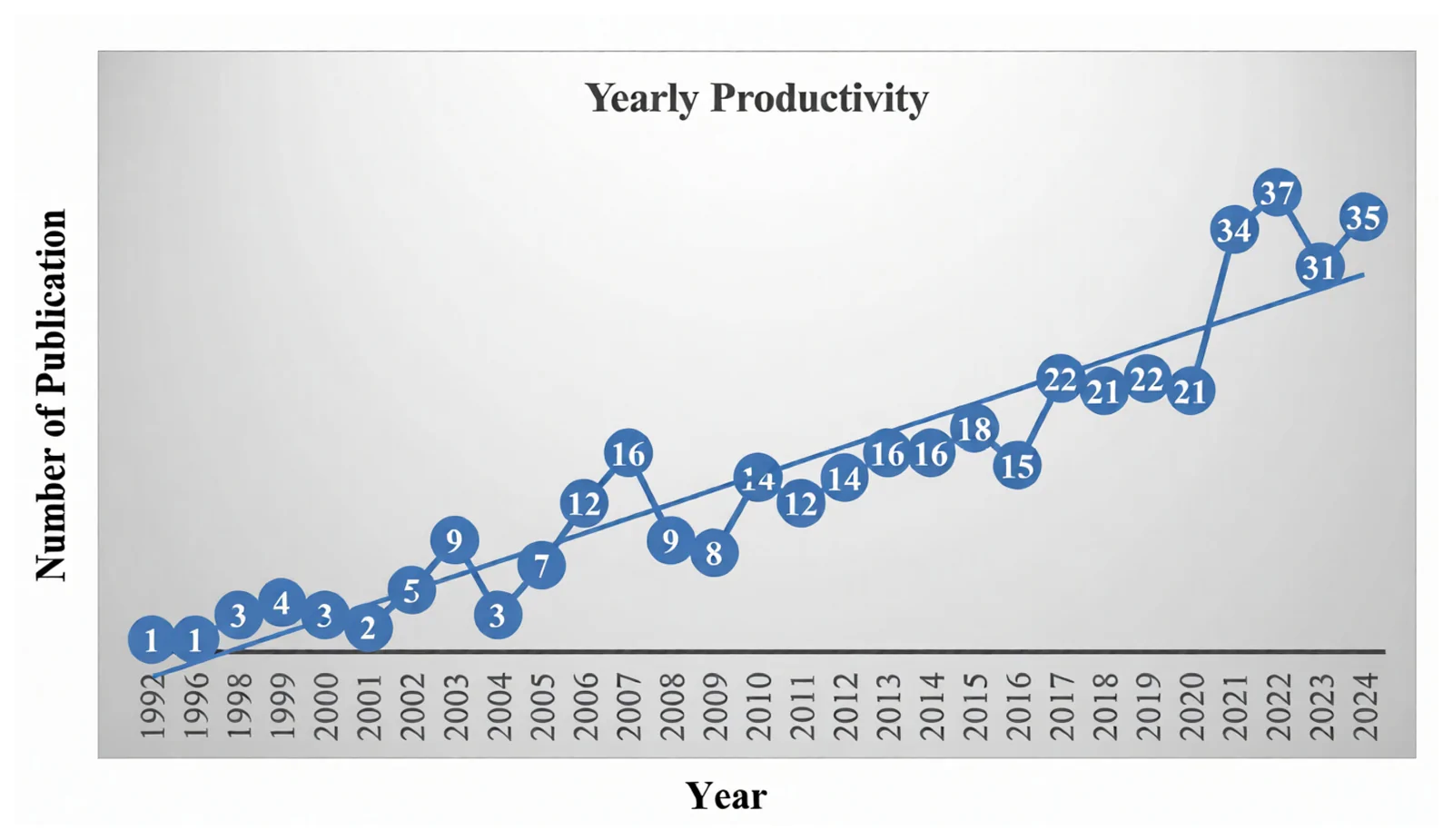
Yearly growth in publications.

Sections 4.2 and 4.3, which describe the document types of publications related to the topic (Fig. S2, Supplemental Digital Content 3) and present the authorship patterns (Fig. S3, Supplemental Digital Content 4), are available in File S1, Supplemental Digital Content 9.

### 4.2. Most productive authors

Table 1 highlights the most productive and influential authors, presenting data on TP, total citations (TC), C/P, and their active research years.

**Table 1:**
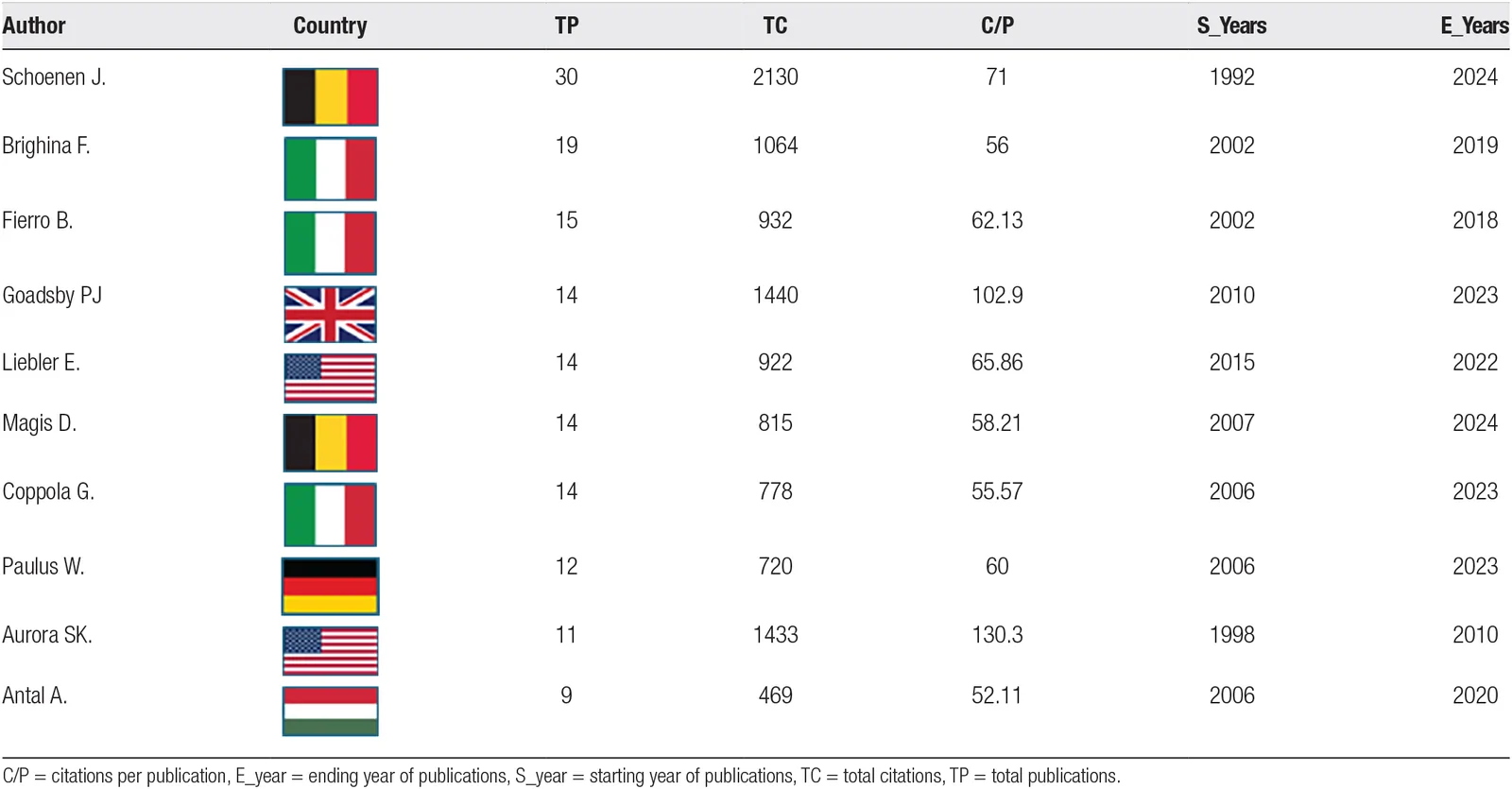
Most productive authors.

The analysis ranked Schoenen J. from Belgium at the top position with the highest number of documents (n = 30) and 2130 TC, averaging 71 C/P. His long active period from 1992 to 2024 reflects a sustained and influential presence in the field. Following that, 2 Italian authors, Brighina F. and Fierro B., were at the second and third positions with 19 and 15 documents, respectively. On the other hand, Aurora SK. has the highest C/P ratio of 130.3, despite a lower publication count (11 documents) and a shorter active period (1998–2010), which indicates exceptionally high-impact work. Goadsby PJ. (C/P: 102.9) and Liebler E. (C/P: 65.86) also show high influence relative to their publication numbers, suggesting that their contributions are widely recognized and frequently referenced. Moreover, Fierro B. (C/P: 62.13) and Paulus W. (C/P: 60.0) show a strong balance of productivity and impact.

Authors like Schoenen J., Magis D., and Coppola G. show ongoing research activity into 2024, indicating current engagement and possibly leadership roles in ongoing NIBS studies. However, others, such as Fierro B. and Aurora SK., have earlier end years (2018 and 2010, respectively), suggesting that while their contributions are foundational, they are no longer actively publishing in this area.

In summary, this analysis reveals a group of core researchers who have driven the evolution of NIBS-based migraine research through both volume and scholarly impact. Schoenen J. leads in total output, while Aurora SK. and Goadsby PJ. stand out for the influence of their individual publications. The continued involvement of several authors into 2024 might indicate progress and leadership in this research area.

### 4.3. Most productive organizations

Table 2 summarizes the top 10 prolific organizations publishing research related to the topic, highlighting TP, TC, and the span of publication years.

**Table 2:**
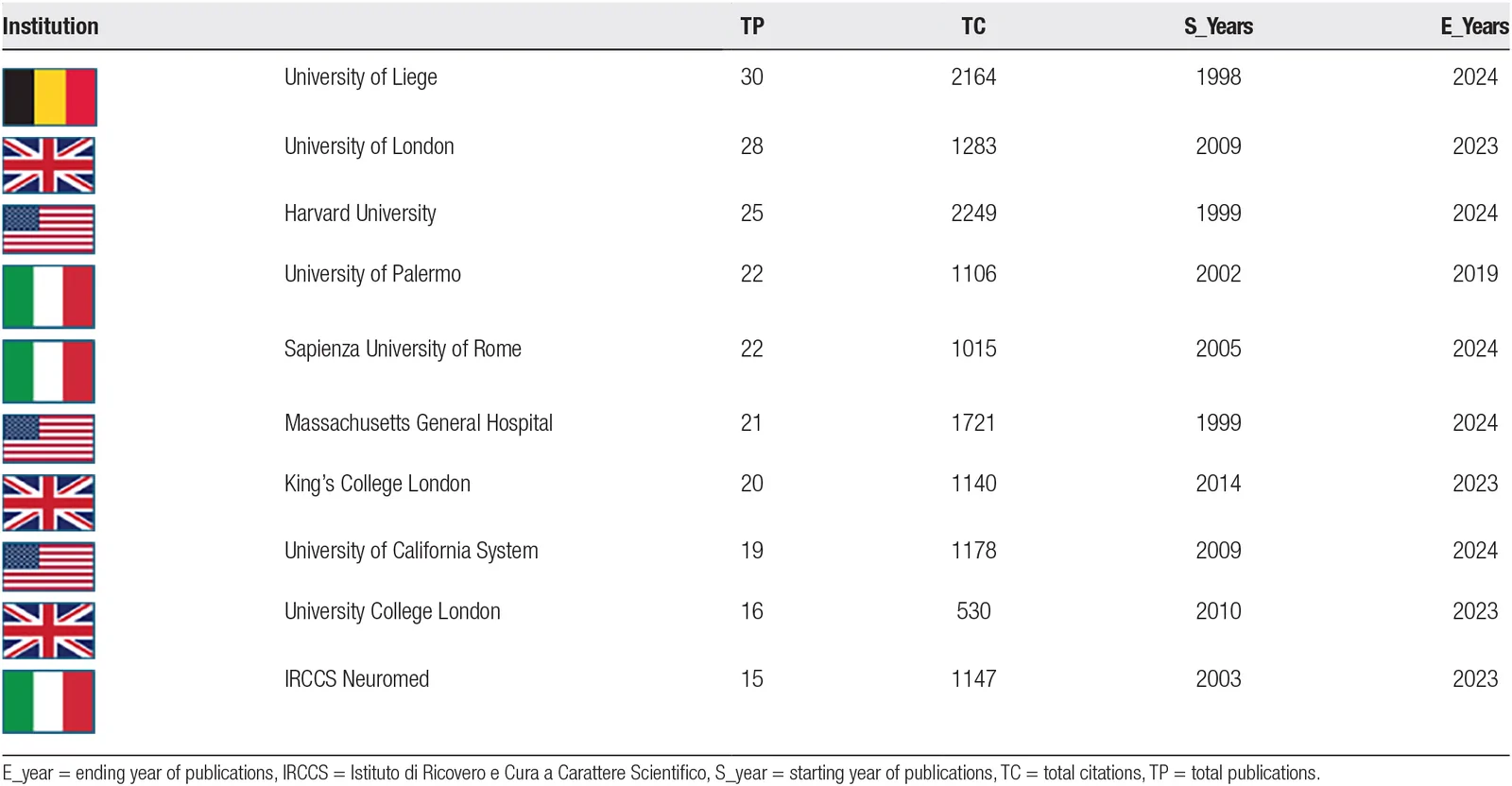
Most productive organizations.

The University of Liège (Belgium) leads with 30 publications and 2164 citations, indicating both sustained productivity and strong academic influence over a 26-year span (1998–2024). The University of London and Harvard University follow with 28 and 25 publications, respectively. While London’s output spans 14 years (2009–2023), Harvard shows higher impact with 2249 citations over a 25-year period (1999–2024). The University of Palermo and Sapienza University of Rome both maintained the fourth position due to publishing an equal number of publications. Istituto di Ricovero e Cura a Carattere Scientifico Neuromed also contributed significantly with 15 publications and 1147 citations, reflecting high C/P performance.

Harvard University stands out with the highest TC, reflecting significant impact despite not being the most prolific. Massachusetts General Hospital, affiliated with Harvard, also shows a strong citation record (1721 citations from 21 publications), supporting the high research caliber of the Harvard-Massachusetts General Hospital network.

As emerging and recent contributors, King’s College London and University College London show recent but impactful activity, with their publications concentrated in the last decade. Furthermore, the University of California System (19 publications, 1178 citations) illustrates growing US-based institutional engagement in the field since 2009.

### 4.4. Most productive countries

Table 3 presents the top 10 countries contributing to the field of NIBS for migraine, based on TP, TC, C/P, and publication time span.

**Table 3:**
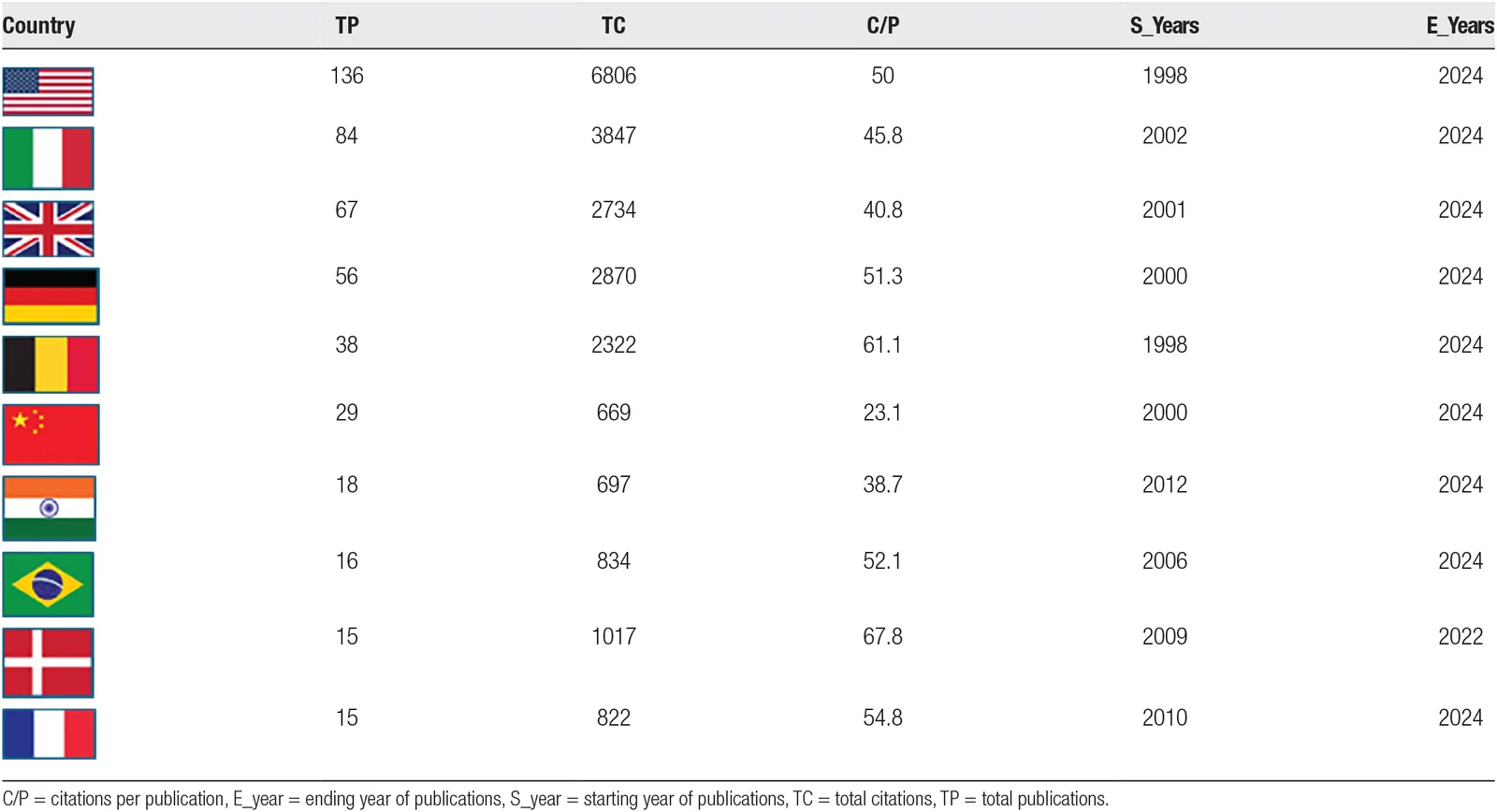
Most productive countries.

The analysis showed that the US is the most productive country with 136 publications and the highest TC (6806). Italy and the United Kingdom (UK) follow, with 84 and 67 publications respectively, showing strong and notable research activity. Denmark has the highest C/P rate (C/P: 93.1), suggesting a small but highly impactful research output. Belgium and Germany exhibit high citation impact (C/P: 61.1 and 51.3), indicating influential research despite fewer papers.

In summary, the field of NIBS migraine research is driven by a combination of high-output countries like the US, high-impact contributors like Denmark, and collaborative hubs like Belgium and the Netherlands. The US and Europe dominate the landscape, with emerging contributions from countries like China, India, and Brazil.

### 4.5. Most prolific sources

Table 4 describes the most prolific sources publishing research related to the topic under study. The analysis revealed that out of the top 10 most prolific sources, 7 belonged to the first quartile (Q1), and only 3 belonged to the second quartile (Q2). This indicates that the top 10 most prolific sources are also high-quality journals. They are both highly productive and high-quality journals. The analysis ranked the journal Cephalalgia at the top with 42 publications, followed by Headache with 30 publications, and the Journal of Headache and Pain with 26 publications. The citation-wise analysis also ranked Cephalalgia in the top position with 2516 citations, followed by Headache with 1960 citations, and the Journal of Headache and Pain with 1233 citations. However, the journal Neurology obtained the highest C/P, followed by the journal Headache and the journal Pain. The figure also presents the starting and ending years of publications by these sources.

**Table 4:**
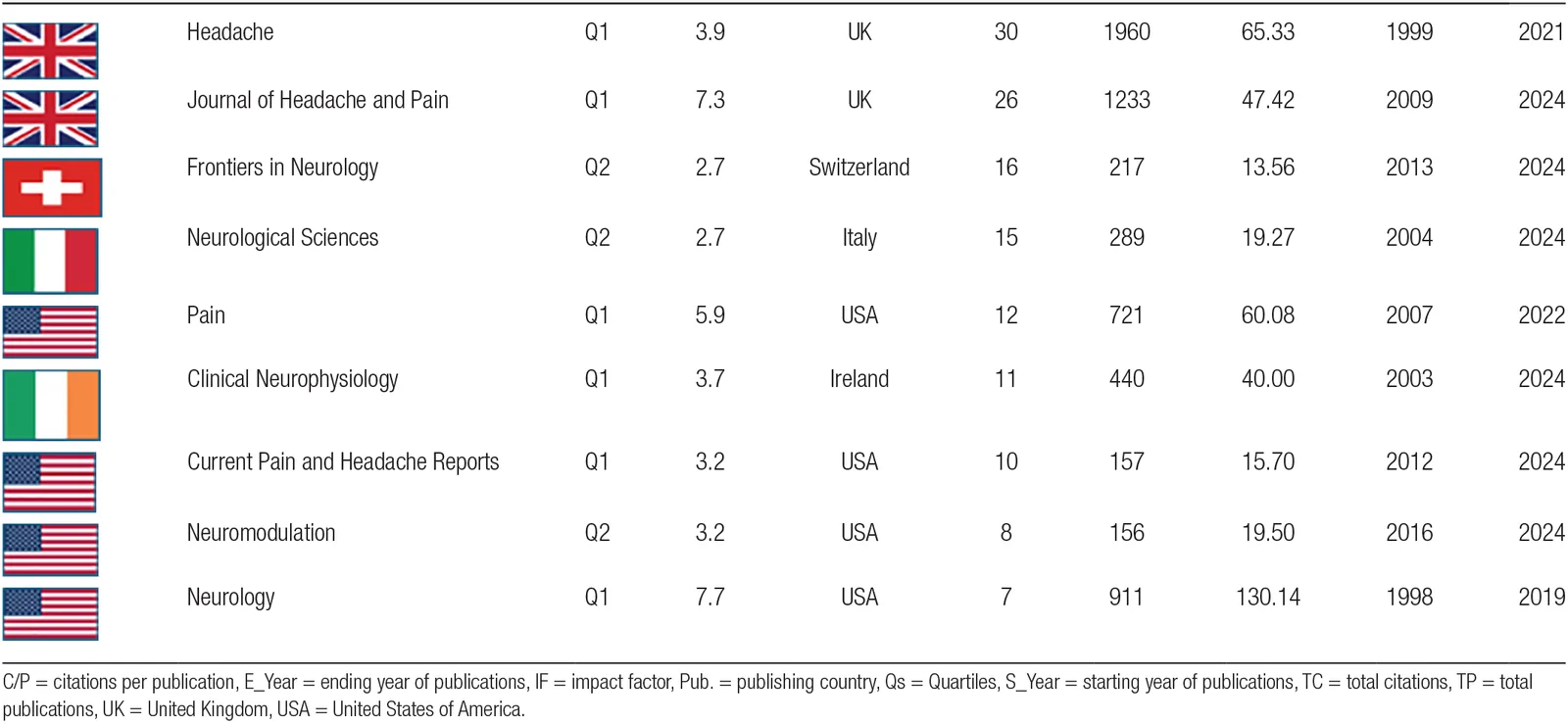
Most prolific sources.

### 4.6. Top 10 keywords with the strongest citation bursts

Citation-bursts highlight keywords that experience a sudden, rapid increase in citations during a specific period, pointing out the dynamics, emerging frontiers, and directional trends of a research area. Figure 3 illustrates the top 10 keywords with the strongest citation bursts, spanning from 1996 to 2024. By analyzing the temporal distribution of these bursts, 3 distinct evolutionary phases of the research field emerge. First, the foundational pathophysiology phase (1996–2010) was dominated by terms such as aura (1996–2005), hyperexcitability (1999–2008), and occipital cortex (1999–2010), reflecting early efforts to understand basic neurophysiological mechanisms. Second, a methodological expansion occurred between 2000 and 2014; notably, while TMS exhibited the highest overall burst strength (11.61) from 2001 to 2008, the keyword cortical excitability demonstrated the most sustained research interest, maintaining a strong citation burst over the longest period (2000–2014). This era also saw an intense focus on neurophysiological assessment tools, highlighted by bursts in intensity dependence and evoked potentials (both 2003–2011). Finally, a recent translational and clinical shift is evident from 2017 to 2024. The emergence of prevention (2017–2024) and prophylactic treatment (2019–2021, strength: 10.01) as the most active recent frontiers indicates a clear pivot in the literature from basic science and diagnostics toward proactive clinical management. Ultimately, this temporal evolution underscores the field’s maturation, with current research trajectories heavily favoring preventive clinical interventions.

**Figure 3.**
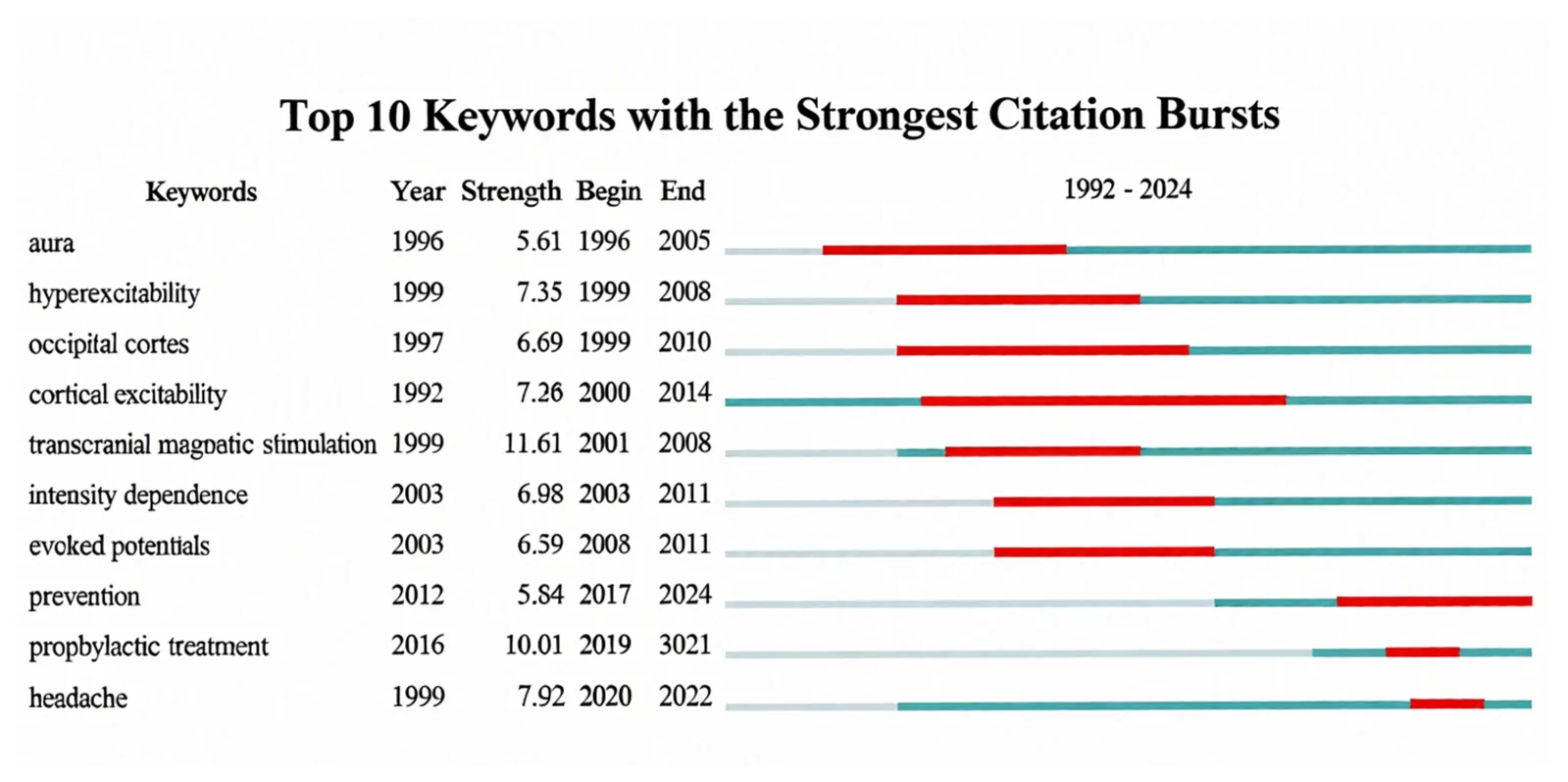
Top 10 keywords with the strongest citation bursts (1992–2024). This figure illustrates the temporal evolution and intensity of emerging research frontiers in the field. “Year” indicates the first appearance of the keyword, “Strength” denotes the burst intensity (magnitude of the citation surge), and “Begin” and “End” represent the start and end years of the active burst period.

### 4.7. Co-occurrence of author keywords

Figure 4 describes the co-occurrence of author keywords. It reports on the author keywords and how frequently they appear together in the same article, and how they relate to each other. The graph divides the keywords into 6 clusters represented by different colors. The keywords have been thematically grouped in the clusters, showing the area of research that is strongly connected. Cluster 1 in green, consists of keywords such as Migraine, Headache, Brain Stimulation (TMS, tDCS), Cortical Excitability, Aura, and Inflammation. Cluster 2, in blue, consists of Chronic Migraine, Migraine with/without Aura, and Depression. Cluster 3 in orange, includes Cluster Headache, Chronic Headache, Vagus Nerve, and Neurostimulation. Cluster 4 in purple, consists of keywords like Systematic Review and Meta-Analysis. Cluster 5 in red, includes keywords like Episodic Migraine and Treatment. Cluster 6 in pink, includes Calcitonin Gene-Related Peptide.

**Figure 4.**
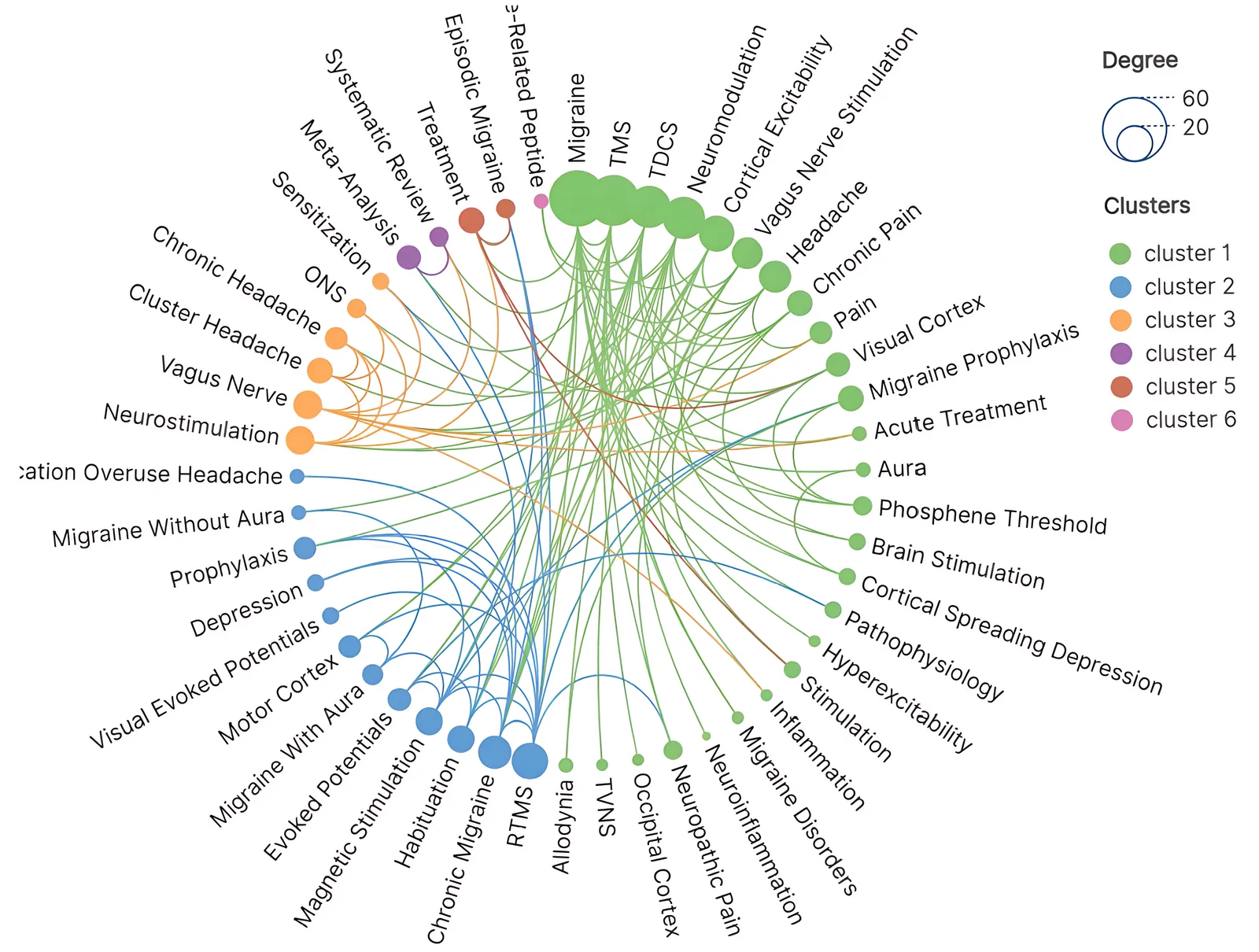
Co-occurrence of author keywords. Node size represents keyword occurrence frequency, whereas node color denotes membership in the corresponding keyword cluster. Lines represent co-occurrence relationships between keywords, with greater link strength indicating stronger co-occurrence. ONS = occipital nerve stimulation, RTMS = repetitive transcranial magnetic stimulation, TVNS = transcutaneous vagus nerve stimulation.

Sections 4.10, 4.11, 4.12, and 4.13, presenting the following: a word cloud of author keywords (Fig. S4, Supplemental Digital Content 5), co-authorship density visualization (Fig. S5, Supplemental Digital Content 6), evolution of research by subject category (Fig. S6, Supplemental Digital Content 7), and co-citation network of the documents (Fig. S7, Supplemental Digital Content 8), are available in File S1, Supplemental Digital Content 9.

### 4.8. Countries’ collaboration patterns

Figure 5 illustrates the international collaboration network among countries. In this network visualization, each node represents a country, with node size proportional to the number of publications, and the connecting lines indicate collaborative relationships between countries. The thickness of the lines reflects the strength of collaboration. The analysis reveals that the US, Germany, UK, and Italy emerged as the most prolific and collaborative nations, serving as central hubs in the global research network. This finding aligns with the historical leadership of these countries in migraine neurophysiology and neuromodulation research. Notably, European countries demonstrate robust intercontinental collaboration, forming dense clusters that reflect strong regional research partnerships. In contrast, countries such as Afghanistan, Ukraine, and Peru appear as peripheral nodes with limited collaborative output, likely reflecting constraints in research infrastructure and funding. While Asian countries including China, Japan, South Korea, and Iran are represented in the network, their collaborative ties appear less extensive compared to European nations, suggesting opportunities for enhanced international partnerships to advance the field globally.

**Figure 5.**
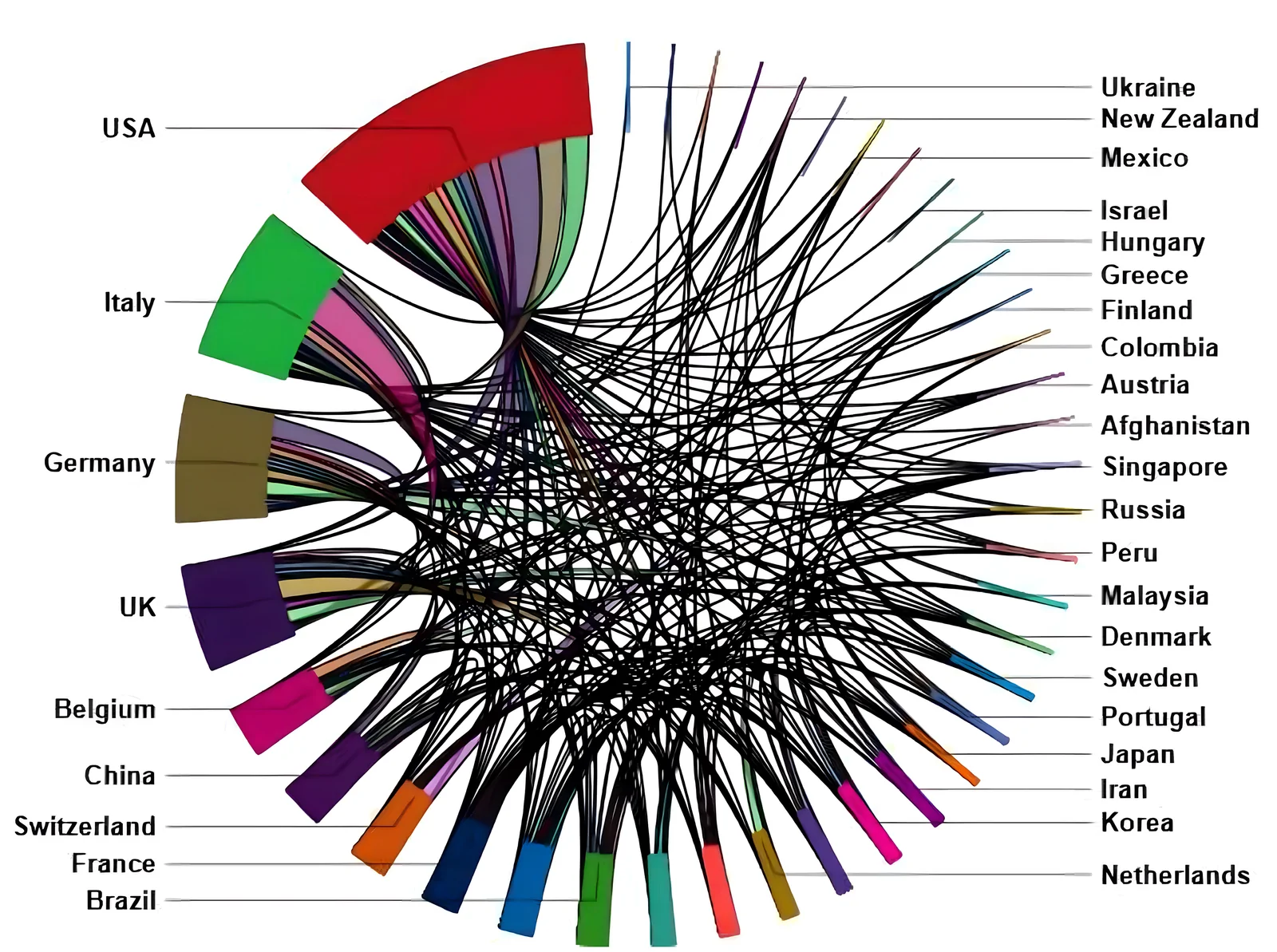
Network visualization of international collaboration. Each node represents a country, with the node size proportional to its total publication output. The connecting lines indicate co-authorship relationships between 2 countries, and the thickness of the lines reflects the strength (frequency) of the collaboration. UK = United Kngdom, USA = United States of America.

The prominence of the US and several European countries within the collaboration network may reflect the presence of established headache and neuromodulation research centers, sustained research activity, and well-developed international academic networks. These factors should be considered potential contextual explanations rather than causal determinants, as the present bibliometric analysis did not directly evaluate funding, institutional resources, or research infrastructure.

### 4.9. The correlation between the number of references (NR) cited and the TC obtained

The study analyzed the correlation between the NR cited and the TC obtained by the articles. A Pearson correlation coefficient was calculated to assess the association between these variables. The analysis showed a strong positive correlation between the NR cited and TC obtained (*R* = 0.88, *P* < .01). This result was interpreted as an association rather than a causal relationship.

## 5. Discussion

This study provides a comprehensive bibliometric overview of research on NIBS for migraine, revealing a field that has evolved substantially over the past 3 decades. The observed increase in publications reflects an expansion in scientific output and a broader development of neuromodulation research in migraine. The accelerated growth in publications after 2009 coincided with several developments, including increased recognition of cortical hyperexcitability as a mechanism relevant to migraine pathophysiology, growing interest in non-pharmacological approaches, and the introduction of clinically applicable neuromodulation devices.^[2,20]^ The FDA approval of sTMS for migraine with aura in 2013 also occurred during this period of increasing research activity.^[10]^ However, the temporal coincidence between regulatory developments and publication growth should not be interpreted as evidence that the approval directly caused the increase in research output. The randomized controlled trial by Lipton et al^[21]^ and subsequent studies such as Silberstein et al^[22]^ provided important clinical evidence concerning sTMS, but the present bibliometric analysis does not directly evaluate therapeutic effectiveness or clinical adoption.

The marked increase in publication activity after 2009 also occurred during a period of important clinical and regulatory developments in migraine neuromodulation. In particular, the 2013 US FDA approval of sTMS for migraine with aura represented an important regulatory milestone.^[10]^ The temporal association between this milestone and subsequent publication growth is noteworthy; however, the present bibliometric analysis cannot establish that regulatory approval directly caused the increase in research output. Rather, the observed pattern may reflect the combined influence of accumulating clinical evidence, technological development, increasing scientific interest, and regulatory recognition of neuromodulation.

Importantly, several of the most highly cited articles identified in the present bibliometric analysis laid the mechanisms and clinical foundations of the field. The pioneering studies by Aurora and colleagues demonstrated that patients with migraine exhibit increased occipital cortical excitability and lower phosphene thresholds, providing the first direct physiological evidence that cortical hyperexcitability may predispose individuals to cortical spreading depression and migraine aura.^[23,24]^ These studies shifted the scientific focus from purely vascular theories of migraine toward neurophysiological mechanisms and established cortical excitability as a feasible therapeutic target for neuromodulation.

The transition from theoretical understanding to clinical practice was further accelerated by the randomized controlled trial by Lipton et al,^[21]^ one of the most influential and highly cited studies in this field. This study demonstrated that sTMS improved pain freedom and reduced associated migraine symptoms in patients with migraine with aura.^[13]^ The temporal proximity of this clinical evidence, subsequent regulatory developments, and the increase in publication activity identified in our bibliometric analysis is noteworthy. However, the present analysis cannot determine whether these events directly caused the subsequent increase in scientific output. The continued influence of this work is evident from the sustained citation growth and the emergence of TMS as the dominant keyword cluster within the literature.

Similarly, the highly cited contributions of Goadsby and colleagues expanded the conceptual and therapeutic research landscape of migraine neuromodulation.^[20,25,26]^ Their work highlighted the importance of noninvasive neuromodulation approaches, including VNS, and emphasized migraine as a complex brain network disorder involving multiple interconnected pain-processing systems. These contributions provide contextual background for the emergence of keywords such as “vagus nerve stimulation,” “neurostimulation,” and “chronic migraine” in our thematic analyses. The bibliometric findings indicate increasing scientific interest in these topics, but they do not establish that specific conceptual advances directly caused the observed keyword trends.

Collectively, the seminal studies by Aurora, Lipton, Goadsby, and their colleagues represent 3 distinct but interconnected phases in the evolution of the field: identification of abnormal cortical excitability as a pathophysiological substrate, translation of this knowledge into clinically effective TMS interventions, and expansion toward multimodal and personalized neuromodulation strategies for migraine management.

Consistent with these seminal contributions, the bibliometric dominance of TMS and tDCS is unsurprising given the substantial evidence supporting their therapeutic potential. Both techniques are directly linked to the modulation of cortical excitability, which has long been considered a hallmark of migraine pathophysiology.^[27]^ Early TMS studies proposed that magnetic stimulation of the occipital cortex could interrupt cortical spreading depression, the electrophysiological phenomenon believed to initiate migraine aura and activate trigeminovascular pain pathways.^[13]^ Similarly, tDCS has demonstrated the ability to induce long-lasting changes in cortical excitability and synaptic plasticity, potentially reducing headache frequency and migraine-related disability.^[8,9]^ These findings are further supported by recent systematic reviews and meta-analyses showing the therapeutic efficacy of TMS and tDCS across several neurological and neuropsychiatric disorders.^[6]^ Therefore, the prominence of TMS and tDCS in our bibliometric analysis likely reflects both their strong mechanistic foundations and their relatively mature clinical evidence base.

Several limitations should be considered when interpreting the present findings. First, the analysis was restricted to the WoSCC. Although WoSCC provides structured bibliographic and citation information suitable for bibliometric analysis, publications indexed exclusively in Scopus, PubMed, Embase, or other databases may have been missed, introducing potential database-selection bias. Second, the database may underrepresent some non-English publications, resulting in potential language bias. Third, citation-based indicators are inherently influenced by publication age, as older studies have had more time to accumulate citations. Citation counts may also be affected by self-citation, field-specific citation practices, and differences in citation behavior across research areas. Fourth, recent publications may be underrepresented in citation-based analyses because of delays between publication, database indexing, and citation accumulation. Fifth, country productivity was assigned according to the first-listed country for publications with multiple affiliations. Although this approach provides consistent single-country attribution, it may underestimate the contribution of secondary-affiliation countries and, therefore, simplify international research contributions. Sixth, assigning publications with multiple Web of Science subject categories to the first-listed category may similarly oversimplify multidisciplinary research. Finally, bibliometric indicators measure publication activity, citation patterns, and research connectivity rather than clinical effectiveness. Consequently, the present findings should not be interpreted as evidence of therapeutic efficacy, clinical superiority, or causal relationships between specific events and changes in research output. Similar limitations have been highlighted in evidence-based guidelines and comprehensive reviews of therapeutic brain stimulation techniques.^[3,26,28]^

Overall, the present bibliometric findings suggest that research on NIBS for migraine is entering a phase characterized by technological diversification, continued clinical interest, and expanding opportunities for mechanistic and personalized research. Future work may benefit from improved mechanistic understanding, standardized methodologies, stronger international collaboration, and broader geographic representation. These priorities are derived from the publication, collaboration, and thematic patterns identified in the present analysis and should not be interpreted as evidence of therapeutic efficacy or established clinical adoption.

## 6. Conclusion

The present bibliometric analysis characterized the development and intellectual structure of research on NIBS for migraine from 1992 to 2024. Publication activity increased substantially over the study period, with the US, Italy, and the UK among the most productive countries and several European countries showing prominent collaboration patterns. TMS and tDCS represented established research themes, while VNS emerged as a prominent recent research frontier. The thematic and keyword analyses further demonstrated a shift from early emphasis on cortical excitability and migraine pathophysiology toward clinical subtypes and increasingly diverse neuromodulation approaches.

These findings provide a quantitative overview of publication trends, research collaboration, citation patterns, and evolving research priorities in the NIBS–migraine literature. The identified geographic and thematic gaps may help guide future bibliometric, experimental, and clinical research. However, because bibliometric methods assess scientific publication patterns rather than therapeutic efficacy, the present findings should not be interpreted as evidence of clinical effectiveness or causal relationships.

## Author contributions

**Conceptualization:** Turki Abualait, Mohamed A. Al Maghraby.

**Data curation:** Shahid Bashir, Muhammad Ajmal Khan, Nadeem Siddique.

**Formal analysis:** Shahid Bashir, Muhammad Ajmal Khan, Nadeem Siddique.

**Investigation:** Turki Abualait.

**Methodology:** Muhammad Ajmal Khan, Nadeem Siddique, Suzan Ahmed Al-Hamadah, Saliha Mohammed Alaali, Zahra Abdulmuttalib Al-Muhanna, Danah Khalid Al-jaafar, Lamia Ali Al-salman, Rama Emad Shabana, Zahra Hadi Alqallaf.

**Project administration:** Turki Abualait, Mohamed A. Al Maghraby.

**Software:** Muhammad Ajmal Khan, Nadeem Siddique.

**Supervision:** Shahid Bashir, Mohamed A. Al Maghraby.

**Validation:** Turki Abualait, Shahid Bashir, Muhammad Ajmal Khan, Nadeem Siddique.

**Visualization:** Turki Abualait, Shahid Bashir, Muhammad Ajmal Khan, Nadeem Siddique.

**Writing – original draft:** Turki Abualait, Mohamed A. Al Maghraby, Suzan Ahmed Al-Hamadah, Saliha Mohammed Alaali, Zahra Abdulmuttalib Al-Muhanna, Danah Khalid Al-jaafar, Lamia Ali Al-salman, Rama Emad Shabana, Zahra Hadi Alqallaf.

**Writing – review & editing:** Turki Abualait, Mohamed A. Al Maghraby, Suzan Ahmed Al-Hamadah, Saliha Mohammed Alaali, Zahra Abdulmuttalib Al-Muhanna, Danah Khalid Al-jaafar, Lamia Ali Al-salman, Rama Emad Shabana, Zahra Hadi Alqallaf.


















