# Supplementary material for: Noninvasive brain stimulation for migraine: A bibliometric analysis of scientific research with thematic and keyword mapping
Source: Medicine (Baltimore). 2026 Oct 30;105(44):e51349. doi: 10.1097/MD.0000000000051349 (PMC13727236; doi:10.1097/MD.0000000000051349)
Supplement: Supplementary file 1 [file medi-105-e51349-s001.docx]

**Supplementary Data**

Supplementary Figure 1 provides a summary of the dataset. It discloses the range of publishing years of the research that starts from 1992 and ends in 2024. A total of 411 documents were published in this time span in a total of 159 sources. It indicates 11.75% as the annual growth rate of the documents. A total of 1587 authors were involved in the outcoming 411 documents. There were 21 authors who conducted research on the topic as single authors. There was 29.93% of work that involved international collaboration. The summary also shows that there were 5.72 co-authors per document. There were a total of 800 author-supplied keywords, and 12875 references were cited. The average age of the documents was 9.32 years. The average citation per document was 39.18.

4.2 **Document types**

Supplementary Figure 2 describes the document types of the publications related to the topic. The document type ‘Article’ appeared as the most favorite of the researchers for sharing their research (n=287), followed by ‘Review’ with 107 documents, and ‘Article; Proceedings Paper’ with eight documents. The document type ‘Article’ also obtained the highest number of citations (n=11324), followed by ‘Review’ with 4532 citations and ‘Article; Proceedings Paper’ with 240 citations.

4.3 **Authorship pattern**

Supplementary Figure 3 presents the authorship pattern, which ranges from single to 22 authors. The data in the figure reveals a trend of collaborative research, with the highest number of publications contributed by a four-author pattern, followed by two-authored publications and five-authored publications. The three-author and six-author patterns contributed an equal number of publications and ranked in the fourth position. However, 21 publications were contributed by the single-author pattern. The data also reports one publication by the twenty-two-author pattern.

4.10. **Word cloud of author keywords**

Supplementary Figure 4 presents a word cloud of author keywords. The word size represents the frequency of the use of keywords by the authors. The keyword Migraine emerged as the most frequently used keyword by the authors. Likewise, the keyword TMS emerged as the second most frequently used keyword, followed by tDCS and vagus nerve stimulation. The cloud also presents the other frequently used keywords by the authors.

4.11. **Co-authorship density visualization**

Supplementary Figure 5 provides a co-authorship density visualization. The labels in the figure present an author. Likewise, the color and intensity of the area show how frequently the authors collaborate with each other. The red area depicts high collaborative activities in terms of the number of publications or collaborations. Whereas the yellow area indicates a lower level of such activities. The authors who have been placed closer to each other show their most frequent collaborative activities. This type of visualization is mostly used to recognize the collaborative patterns among the researchers in a given field of study. It also helps in identifying the key active researchers in that particular field of study. It also helps aspiring researchers who want to collaborate with other researchers in their fields of study. The analysis presents researchers like Brighina, Filippo, Fierro, Brigida, Liebler, Eric, De Tommaso, Marina, and Barbanti, Piero, as the key collaborative researchers in this field of study.

4.12. **Subject categories-wise evolution of research**

The following visualization (Supplementary Figure 6) was generated with the help of a visualization tool, CiteSpace. This type of visualization helps to provide declining or emerging trends and to understand research focus with the passage of time. The X-axis provides the timeline, which is from 1996 to 2024. Likewise, the Y-axis provides the Subject categories. The bump in the graph shows any research activity at the specific time. Whereas the height and width of the bump present the duration and volume of the research activity. The graph indicates that the subject category Psychology had research activities from 1996 to 2024, and Neuroscience had its peak in research activities from 2003 to 2016. Likewise, the graph presents the research activities of other subject categories.

4.13. **Co-citation network of the documents**

Supplementary Figure 7, generated by VOSviewer software, represents clusters of documents that are cited together, indicating related research themes. The figure has nodes that indicate authors and publication years, and color that represents thematic similarity or co-citation frequency. The distance between two nodes shows how frequently the documents are cited together in research. If the documents are closer to each other, it indicates that they are more frequently cited together in the documents. The size of the nodes represents the frequency of the citation or co-citations. A larger size of nodes indicates higher citations or cocitations.
